# Diverse glasses revealed from Chang’E-5 lunar regolith

**DOI:** 10.1093/nsr/nwad079

**Published:** 2023-03-21

**Authors:** Rui Zhao, Laiquan Shen, Dongdong Xiao, Chao Chang, Yao Huang, Jihao Yu, Huaping Zhang, Ming Liu, Shaofan Zhao, Wei Yao, Zhen Lu, Baoan Sun, Haiyang Bai, Zhigang Zou, Mengfei Yang, Weihua Wang

**Affiliations:** Institute of Physics, Chinese Academy of Sciences, Beijing 100190, China; Center of Materials Science and Optoelectronics Engineering, University of Chinese Academy of Sciences, Beijing 100049, China; Institute of Physics, Chinese Academy of Sciences, Beijing 100190, China; Institute of Physics, Chinese Academy of Sciences, Beijing 100190, China; Institute of Physics, Chinese Academy of Sciences, Beijing 100190, China; Center of Materials Science and Optoelectronics Engineering, University of Chinese Academy of Sciences, Beijing 100049, China; Institute of Physics, Chinese Academy of Sciences, Beijing 100190, China; Center of Materials Science and Optoelectronics Engineering, University of Chinese Academy of Sciences, Beijing 100049, China; Institute of Physics, Chinese Academy of Sciences, Beijing 100190, China; Center of Materials Science and Optoelectronics Engineering, University of Chinese Academy of Sciences, Beijing 100049, China; Institute of Physics, Chinese Academy of Sciences, Beijing 100190, China; Qian Xuesen Laboratory of Space Technology, China Academy of Space Technology, Beijing 100094, China; Qian Xuesen Laboratory of Space Technology, China Academy of Space Technology, Beijing 100094, China; Qian Xuesen Laboratory of Space Technology, China Academy of Space Technology, Beijing 100094, China; Institute of Physics, Chinese Academy of Sciences, Beijing 100190, China; Institute of Physics, Chinese Academy of Sciences, Beijing 100190, China; Songshan Lake Materials Laboratory, Dongguan 523808, China; Institute of Physics, Chinese Academy of Sciences, Beijing 100190, China; Center of Materials Science and Optoelectronics Engineering, University of Chinese Academy of Sciences, Beijing 100049, China; Songshan Lake Materials Laboratory, Dongguan 523808, China; Qian Xuesen Laboratory of Space Technology, China Academy of Space Technology, Beijing 100094, China; College of Engineering and Applied Sciences, Nanjing University, Nanjing 210093, China; Qian Xuesen Laboratory of Space Technology, China Academy of Space Technology, Beijing 100094, China; China Academy of Space Technology, Beijing 100094, China; Institute of Physics, Chinese Academy of Sciences, Beijing 100190, China; Songshan Lake Materials Laboratory, Dongguan 523808, China; Qian Xuesen Laboratory of Space Technology, China Academy of Space Technology, Beijing 100094, China

**Keywords:** lunar glasses, glass fibers, amorphous rims, micrometeorite impacts, gentle impact environment

## Abstract

Lunar glasses with different origins act as snapshots of their formation processes, providing a rich archive of the Moon's formation and evolution. Here, we reveal diverse glasses from Chang’E-5 (CE-5) lunar regolith, and clarify their physical origins of liquid quenching, vapor deposition and irradiation damage respectively. The series of quenched glasses, including rotation-featured particles, vesicular agglutinates and adhered melts, record multiple-scale impact events. Abundant micro-impact products, like micron- to nano-scale glass droplets or craters, highlight that the regolith is heavily reworked by frequent micrometeorite bombardment. Distinct from Apollo samples, the indigenous ultra-elongated glass fibers drawn from viscous melts and the widespread ultra-thin deposited amorphous rims without nanophase iron particles both indicate a relatively gentle impact environment at the CE-5 landing site. The clarification of multitype CE-5 glasses also provides a catalogue of diverse lunar glasses, meaning that more of the Moon's mysteries, recorded in glasses, could be deciphered in future.

## Teaser

There are diverse glassy materials transforming from liquid, vapor and solid in the Chang'E-5 lunar soils. Particularly, the discovery of ultra-elongated glass fibers and ultra-thin deposited amorphous rims reveals a gentle impact environment of the landing site.

## INTRODUCTION

Lunar glasses are an important component of lunar soils produced by various kinds of non-equilibrium processes on the Moon, including volcanic activities, meteorite bombardment and solar-wind irradiation [1]. These drastic processes dominate the formation and evolution of the Moon, and our understanding of the Moon strongly relies on studies of these processes [1,2]. As ubiquitous products of lunar non-equilibrium processes, glasses can remain stable for billions of years [3]. These glasses with different origins can therefore record crucial information about their formation processes over geological timescales [4–8], and provide insights into many fundamental questions about the Moon relating to the duration of volcanism [9–11], the bombardment history of the solar system [2,5], the origins of lunar water [4,12,13] and the Moon's past magnetism [14].

The most common route to form glasses is the fast cooling of melts. Both volcanism and meteorite impacts on the Moon can produce massive melts [1,6,7]. Glasses produced by volcanic eruptions that have the same age as the period of volcanism could give valuable insights into the thermal and chemical evolution of the Moon's interior [4,6,10,11,15]. For example, volcanic glasses have been found to be rich in water, implying a wet early Moon and challenging the conventional notion of a volatile-poor Moon [4]. The moment the erupted fresh bedrock is exposed to the lunar surface, it begins to receive continuous meteorite impacts, most of which are energetic enough to melt lunar rocks, producing various impact-related glasses [7]. Impacts are the most active processes on the lunar surface [1,2,13,16–20], shaping cratered surfaces [17], developing weathered lunar regolith [18], altering grain surfaces [19,20] and delivering space materials (e.g. water, carbon and other volatiles) to the Moon [13,15,21]. Impacts on the Moon also provide information about the impact history of the solar system [22,23], which is involved with the evolution of planetesimals and terrestrial life [5,15,21]. Meanwhile, impacts on the Moon are highly heterogeneous in both temporal and spatial scale. The striking meteorites can vary from submicron- to kilometer-scale in size, with a varied velocity of 3–50 km/s. Understanding such complex impacts in a long history strongly relies on impact-related glasses [1,2,5,7]. Characterizations of the morphologies, microstructures, compositions and ages of these glasses can help reveal many mysteries, from local impact environment [7,16,17], regional geology and lunar surface water distributions [12,13,15,24], to impact history in the solar system [2,5]. Besides liquid quenching, it is also possible for vapor deposition by micrometeorite impacts [8,19,25–27] and ion implantation by solar-wind irradiation [8,27–30] to accumulate glasses as amorphous rims on lunar grains. Amorphous rims can therefore record information about micrometeorite impacts [25,27,31] and solar activities [30,32]. The irradiated rims play an important role in retaining solar materials of He and H elements [33]. The retained ^3^He is a kind of strategic energy resource, while H may react with lunar minerals producing surface water [12,34–36]. The deposited rims also generally bear nanophase iron particles (npFe^0^), which can alter the spectroscopic signatures of planets. This is known as the space weathering effect [18,20,37–39].

Recently, China's Chang’E-5 (CE-5) mission successfully returned ∼1.73 kg of lunar soils from a mare region in northern Oceanus Procellarum [9,40–43]. Compared with previous Apollo and Luna returned samples that have a limited volcanism age range of 3.9–3.0 Ga and cover only ∼4.4% of the lunar nearside surface [40,44], the CE-5 samples are collected from the youngest lunar region, dated to 2.0 Ga, and higher mid-high latitude, allowing the Moon to be studied in an extended spatiotemporal range [9,40–43]. Preliminary characterizations show that the CE-5 samples are mature samples, but have a significantly lower glass content (8.3%–20.0%) than the Apollo samples (25.4%–72.3%), implying quite a different space environment to the Apollo sites [40,45,46]. A systematic identification and microscopic description of the detailed glassy materials is urgently required for the new lunar regolith with abnormal glass content. Meanwhile, considering the pivotal role of glasses in recording the history of the Moon from various aspects, it is imperative to conduct a comprehensive study and clarify the origins of different glasses collected from this new site. However, such a study of glasses in the CE-5 lunar soils is still lacking so far.

In this work, we perform a comprehensive collection and analysis of diverse CE-5 glasses with different textures, categories and origins. On the basis of detailed morphological, microstructural and geochemical characterizations of large amounts of lunar grains, we demonstrate that the CE-5 samples contain a wide variety of glassy or amorphous materials, including glass particles with diverse shapes (globule, ellipsoid, dumbbell and teardrop), glass fibers with large elongations, adhered glasses with different distribution patterns (agglutinate-like, splash-like, droplet-like glasses and ring-like glasses of microcraters), and amorphous rims induced respectively by vapor deposition and irradiation damage. With clarification of the formation mechanism of different glass types, the environment and regolith evolution at the CE-5 landing site are constrained. The irradiated amorphous rims produced by solar wind and the widespread degassing vesicles in adhered glasses indicate the significant role of solar wind in modifying lunar regolith at the mid-high latitude of the Moon. Common micron- to nano-scale impact products (e.g. micron-scale grains, submicron-scale adhered melts and nano-scale craters) collectively reflect frequent micrometeorite impacts. In particular, we find a new type of glass, glass fibers with elongations (the ratio of length to width of an object) larger than 50, in comparison to Apollo grains with elongations less than 10 [1]. Additionally, the observed deposited rims on the CE-5 soils are npFe^0^-free and almost one-tenth as thin as those of Apollo samples. The distinctions indicate a relatively gentle impact environment at the CE-5 landing site. These findings collectively suggest that small-scale and gentle impacts played a pivotal role in reworking the lunar surface at the CE-5 landing site, and such an impact environment can have implications for regolith evolution, water distributions and space weathering of the lunar surface. Besides constraining space environments, the detailed classification of multiple kinds of lunar glasses could serve as a lunar glass catalogue for further extraction of valuable information about the Moon encoded in corresponding glasses.

## RESULTS

### Glass particles

**Figure 1.**
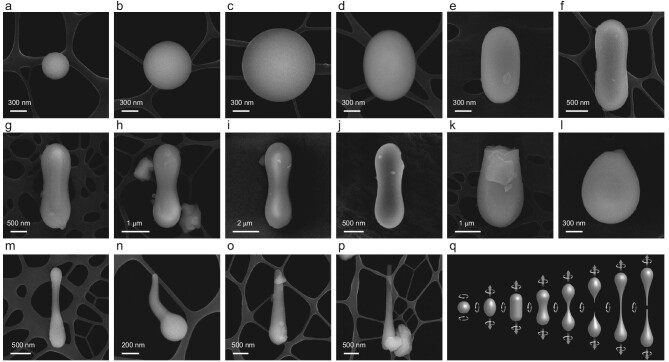
Scanning electron microscope (SEM) images of diverse glass particles. (a–i) Glass globules, oblate spheroids, ellipsoids and dumbbells with different shapes and sizes. (j) Glass dumbbell with a fracture notch on one extremity. (k) Glass dumbbell fractured in half. (l) Fractured glass droplet. (m) Non-centrally thinned glass dumbbell. (n–p) Glass teardrops with different elongations from tadpole and mace to filament shapes. (q) Schematic diagram of the rotation mechanism.

Some typical glass particles, with various shapes and sizes, are firstly shown in Fig. 1 and Fig. S1. The regular glass particles generally have overall rotational shapes, such as globule, oblate spheroid, ellipsoid, dumbbell and teardrop (Fig. 1a–p). Their gross morphologies indicate that they are formed by fast cooling of ejected molten liquid droplets during flight, termed the spray mechanism [47,48]. The amorphous nature of glass particles is further confirmed by the transmission electron microscope (TEM) results, where the TEM images exhibit a typical maze-like pattern and the corresponding fast Fourier transform (FFT) shows diffuse halos (see Fig. S1m–o). The ejected droplets from both volcanic eruptions and impact melting can form globules due to surface tension and low gravity on the Moon [47]. The changing morphologies, through spheroid, ellipsoid, dumbbell and teardrop, suggest that these particles solidify at different stages of rotation. In general, impact glasses are more prone to form such rotational shapes [49,50]. The impacts can give droplets non-axisymmetric rotations about two axes, as illustrated in Fig. 1q. The associated centrifugal force will elongate spherical viscous droplets to form ellipsoids, dumbbells and finally teardrops, as the splitting of dumbbells. Generally, the surfaces of these quenched glass particles are very smooth, exhibiting the character of ‘frozen liquids’. Their surfaces sometimes have the flowing features of viscous liquids or adhered fragments probably arising from micro-impacts, as shown in Fig. S1a–f [46]. Moreover, fractured glass particles are frequently observed, like dumbbells that have notches or are broken in half (Fig. 1j–l and Fig. S1h–l), reflecting a bombardment of micrometeorites.

The provenance of glass particles is further determined from their compositions according to several criteria proposed by previous studies (see Fig. S3) [7,51]. Most of the studied glass particles are thought to be impact glasses for their low MgO/Al_2_O_3_ ratios [51]. Recently, Tarduno *et al.* reported Fe-Ni bearing inclusions that are smaller than 1–5 μm in size in Apollo sample 64 455, a young 2 Ma impact glass [14]. Here, we identified similar Fe-rich metallic inclusions in the interior of a glass particle. In comparison, these inclusions are much smaller, ∼50 nm in size, and contain ∼2% Ni (see Fig. S2). The observed Fe-Ni inclusions are associated with impactor materials, providing a definitive indicator of meteorite impact origins [1,14,52]. Metallic inclusions preserved in impact glasses may also have the potential to record the magnetic field at the moment of collision, which can help us study the Moon's past magnetism [14]. Part of the impact glasses may contain exotic materials that could have been transported by distant giant impacts [44,53]. A small minority of the glass particles seem to have MgO/Al_2_O_3_ ratios >1.25, similar to the picritic volcanic glasses of the Apollo collections [6,51]. But whether they are volcanic glasses needs further confirmation since individual criteria is not completely diagnostic.

### Glass fibers

**Figure 2.**
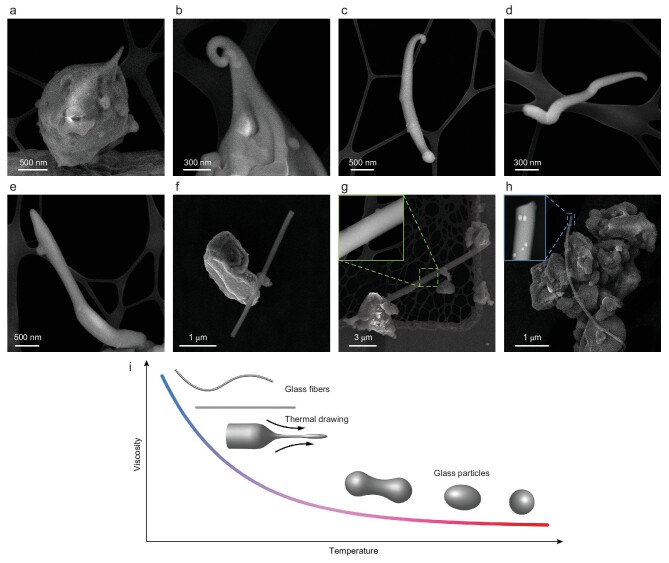
SEM images of various glass fibers. (a and b) Glass filaments drawn from one extremity of the two grains. (c–e) Independent tortuous glass filaments. (f–h) Glass fibers with large elongations. The inset in (g) is the closeup of the marked region. The inset in (h) is the HAADF images of the marked region. The fiber contains dispersed npFe^0^ (bright dots) as typical products of space weathering on the Moon, confirming that the glass fiber is indigenous to the CE-5 soils. (i) Schematic diagram of the relationship between viscosity of melts and morphology of resultant glasses. The filaments and fibers are generally curved or tortuous to store the thermal energy into elastic energy during fast cooling.

Besides diverse glass particles, a new type of glass, glass fibers, are identified in the CE-5 samples. As shown in Fig. 2a and b, there are respective elongated filaments at one extremity of the two agglutinate grains [see Fig. S4 and Fig. 4 for scanning transmission electron microscope (STEM) observations of the two grains]. The uniaxial elongated filaments are probably drawn from viscous melts by ejection force (see Fig. S5a and b for biaxial drawing of the particles). Similarly, independent elongated glass particles could be produced by thermal drawing of impact-generated viscous melts, from tortuous glass filaments in Fig. 2c–e to ultra-elongated glass fibers in Fig. 2f–h. Elongations of the glass fibers with uniform diameters in Fig. 2f–h can reach the value of 25–56. In addition, differently to rotation-induced glass teardrops with tails of decreasing diameter (Fig. 1n–p), thermal drawing and subsequent freezing of viscous melts could result in glass particles with different morphologies, like tortuous shapes (Fig. 2c–e), spindle shapes (Fig. S5a and b), irregular rods (Fig. S5e–h) and uniform fibers (Fig. 2f–h). In our observations, most of the filaments and fibers are curved or tortuous to store the thermal energy into elastic energy during quenching. The high-resolution transmission electron microscope (HRTEM) and high-angle annular dark-field (HAADF)-STEM images of glass fibers (inset in Fig. 2h and Fig. S6e–p) show that they contain npFe^0^ or surface rims that are typical products of space weathering on the Moon, confirming that the glass fibers are indigenous to lunar samples [20,31]. Lunar simulant materials tried to fabricate artificial glass fibers in laboratories for future lunar base construction [54,55]. Our findings directly demonstrate that glass fibers can be produced *in situ* on the Moon, which could inspire space fabrication of glass fibers, such as homogeneous optical fibers and strengthening structural fibers, required by a future lunar base.

Furthermore, the findings of unique glass fibers can also reveal the local impact environment at the CE-5 landing site. Under the near-vacuum environment on the Moon, the final morphology of a liquid-quenched glass grain is not only affected by surface tension and ejection force, but also controlled by the rheological property of the glass forming liquids [47–50]. As illustrated in Fig. 2i, viscosity, which changes greatly with temperature, determines the moldability of the melts. The morphologies of impact-generated glasses can then be used to constrain the initial temperature of impact-generated melts and therefore the impact intensity [56]. Meteorites with the cosmic velocity of the Moon (15–25 km/s) will produce >100 GPa pressure in the central impact region, resulting in several thousand kelvins, which can completely melt any kind of lunar rock [1]. The generated high-temperature melts with low viscosity will be sprayed away, with sufficient fluidity to relax into globules during flight [47]. For decreased impact velocity, the viscosity of melts increases steeply to the value at which the surface tensional force cannot overcome viscosity, making the rotation-elongated shapes preserved [47–50]. With a further decrease in impact velocity, the viscosity of melts can reach a temperature low enough that the highly viscous supercooled liquids undergo continuous thermoplastic flow [57]. In such cases, ultra-elongated glass fibers can be produced via thermal drawing by impact-generated ejection forces. Previous reports on Apollo samples show that the elongations of grains are <10 (usually 1–3) [1], similar to the elongations of glass particles with rotational shapes in CE-5 soils. However, the elongations of lunar glass fibers newly found here can be even larger than 50 (Fig. 2h), indicating that they originate from thermal drawing of highly viscous melts induced by gentle impacts. Therefore, the findings with regard to indigenous ultra-elongated glass fibers suggest that the CE-5 landing region has a gentler impact environment compared with the Apollo landing regions.

### Adhered glasses

**Figure 3.**
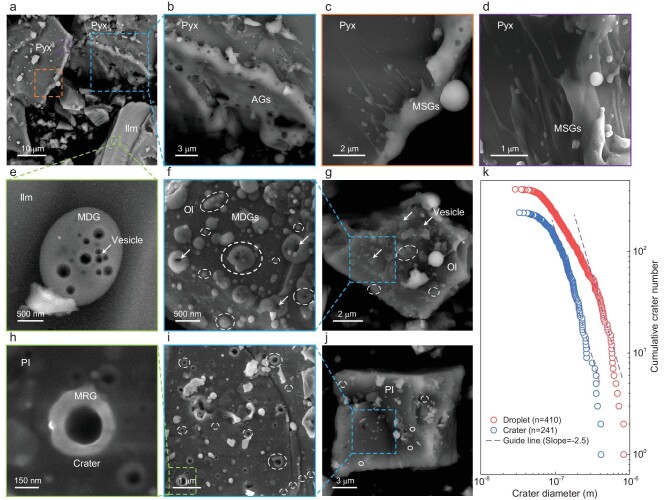
Morphologies of adhered glasses on the surfaces of lunar grains. (a) Back-scattered electron (BSE) image of typical lunar grains with various impact-generated molten features. (b) Close-up BSE image of the top-right pyroxene (Pyx) grain marked in (a). The side of the grain is coated by agglutinate-like glasses (AGs) with vesicles. (c and d) Closeup BSE and secondary electron (SE) images of the two marked regions of Pyx in (a) respectively. The edge of the grain is adhered by molten splash-like glasses (MSGs) with small drops, long tails and flowing features. (e) Isolated molten droplet-like glass (MDG) with rounded degassing vesicles adhered on the surface of the ilmenite (Ilm) grain marked in (a). (f and g) SE and BSE images of densely distributed MDGs exhibiting blistering features adhered on the surface of the olivine (Ol) grain. The circles mark the adhered glass droplets with sizes varying from tens to hundreds of nanometers. Some of the glass droplets are blown up by the degassing gases, as marked by the arrows. (h and i) SE images of spatially clustered craters on the fracture surface of a plagioclase (Pl) grain. There exist raised molten ring-like glasses (MRGs) overhanging the pit edges of the craters. The circles mark the craters with sizes ranging from tens to hundreds of nanometers. (j) BSE image of the fractured plagioclase grain with densely distributed microcraters on the surface. (k) Cumulative size distributions of the 410 glass droplets and 241 craters on the grains in (g) and (j) respectively. The dashed guide lines with a slope of −2.5 mark the steady-state size distributions for the successive collisional fragments.

Another type of liquid-quenched glass is adhered glass, found on certain kinds of CE-5 grains. As shown in Fig. 3a, the shapes of the CE-5 grains are dominated by collisional fractures. There are various micron- and submicron-scale adhered glasses on grain surfaces, with melting and flowing features being evidence of frequent micrometeorite bombardment. Adhered glasses can exhibit as coated agglutinate-like glasses (AGs), shown in Fig. 3b and Fig. S7d and e, where the side of a pyroxene grain is fully coated by agglutinates with vesicular textures. Agglutinates are aggregates of grain fragments cemented by quenched melts generated during repetitive micrometeorite impacts (see Fig. S8 for a typical agglutinate grain) [1,12]. Adhered glasses also commonly exhibit various splash-like shapes, as shown in Fig. 3c and d, Fig. S7b and Fig. S8f. The representative morphologies of these molten splash-like glasses (MSGs) show that a jet of melts crashed into the edge of the host grain at a significant velocity before solidification. Subsequently, the host grain quenched the splashed melts, resulting in MSGs with small drops, long tails and flowing features (see Fig. 3c and d, Fig. S7b and Fig. S8f). An energy dispersive spectroscopy (EDS) line scan shows that compositions of the MSGs and AGs are distinct from their host grains (Fig. S7c and f), suggesting that these glasses are solidifications of exotic ejected melts produced by micrometeorite impacts. Moreover, single and dense molten droplet-like glasses (MDGs) are also found on different grains. As shown in Fig. 3e and Fig. S7g and h, there is a flattened elliptical MDG with rounded vesicles of different sizes adhering on the grain surface. Different to the splashing of melts, isolated MDGs should be the result of the gentle dropping of ejected molten droplets by random single micrometeorite impact events. In addition, densely distributed vesicular MDGs with sizes ranging from submicron- to nano-scale are observed (Fig. 3f and g and Fig. S9), indicating that the grain has experienced a cascade of ejected droplets produced by nearby impacts.

A noticeable feature of the above adhered glasses is that they generally have vesicles of different sizes (Fig. 3b, e–g, Figs S7–9). These vesicles are attributed to the liberation of solar wind gases preserved in lunar grains when molten by micrometeorite bombardment [1,34]. Solar wind is a stem of energetic ions composed mainly of H^+^ (∼95.4%) and He^+^ (∼4.6%). Through long-term irradiation, H^+^ and He^+^ could be implanted and preserved in lunar grains [33,58]. When bombarded by micrometeorites, impact-generated melting will release H and He beneath grain surfaces. Meanwhile, the melting could promote the reduction reaction of H and lunar minerals, producing npFe^0^ and H_2_O [12,34]. Part of H_2_O will be retained, while the rest will be released during melting. Then the liberated gases of H, He and H_2_O could blow up the viscous melts, finally forming the observed degassing vesicles. The common vesicular textures of various adhered glasses show that both micrometeorite impacts and solar wind are involved in the evolution of regolith at the mid-high latitude of the Moon [32], and abundant solar wind materials are preserved in CE-5 soils.

The identified abundant micro-impact products, including widespread AGs, MSGs and MDGs adhered on lunar grains, collectively highlight the important role played by micro-impacts in reworking the lunar surface. The frequent micro-impacts are also reflected by the discovery of microcraters on surfaces of various grains (Fig. S1d, f and g, Fig. 3h–j and Fig. S10). There are discrete or isolated microcraters on some grain surfaces (see Fig. S1d, f and g), indicating random discrete small-scale impacts by nano-scale micrometeorites. Notably, the smooth fracture surface of a plagioclase grain is found to be densely covered with hundreds of microcraters with diameters in the range of 30–400 nm (Fig. 3h–j and Fig. S10). The spatially clustered distribution of these microcraters is probably indicative of secondary impacts [46,59,60]. Meanwhile, the microcraters have similar overall morphologies, suggesting that they were produced by the same impact events [59]. As shown in Fig. 3h and Fig. S10c and d, raised molten ring-like glasses (MRGs) overhanging the pit edges are commonly observed [16]. More importantly, unlike micron-scale craters with spallation zones or surrounding fractured glass filaments in Apollo samples [16,61], the adhered glass rings here are smooth and located tightly around the submicron- to nano-scale craters, indicating a low impact velocity [60,61]. Relative to craters of Apollo samples, the observed glass rings here share more similarity with those of samples from the asteroid Itokawa [61] where the impact velocity is as low as that of the secondary impacts on the Moon [59,61]. Based on these characteristics, it seems reasonable to speculate that these spatially clustered microcraters are secondary craters that are produced by a cascade of debris ejected from a nearby micrometeorite impact. Furthermore, the size distributions of the 410 glass droplets and 241 craters on the grains in Fig. 3g and j are calculated respectively. As shown in Fig. 3k, the cumulative number of droplets or craters varies as a function of droplet or crater diameter. The dashed lines are the power law functions $N( r ) \propto {r}^{( { - D} )}$ with *D* of 2.5 representing the steady-state size distributions for the successive collisional fragments [61,62]. Both the size distributions of droplets and crater are close to the power law functions indicated by the dashed lines, implying that they result from impacts of dusts produced by collision cascade.

### Surface amorphous rims

Distinct from liquid-quenched glasses, glassy materials in the CE-5 soils are also widely found to exist as thin amorphous rims on grain surfaces. Through detailed microstructural and chemical analysis, these amorphous rims are classified as being one of two kinds: micro-impact-generated vapor deposition [8,19,25–27] and solar-wind-caused irradiation damage [8,27–30].

**Figure 4.**
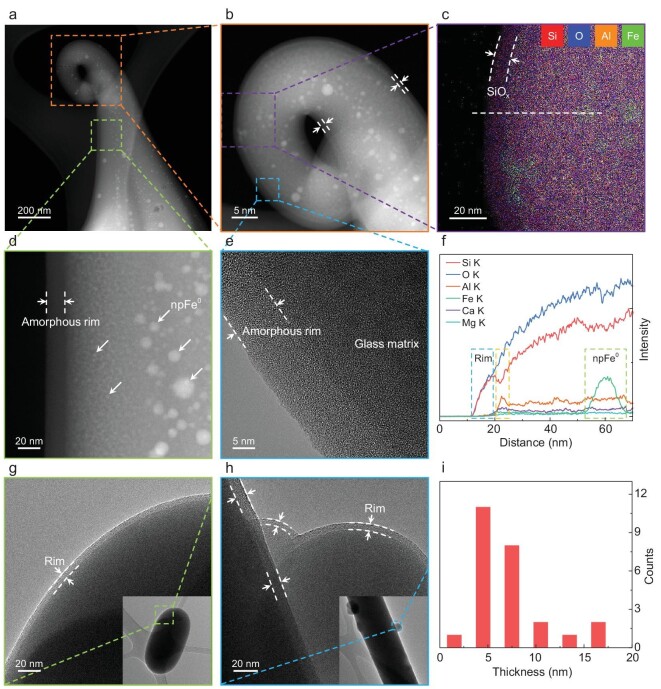
Vapor-deposited amorphous rims. (a and b) HAADF images of the twisted glass filament. There are relatively transparent rims (marked by the double dashed lines) uniformly coating the surface of the grain, and dispersed npFe^0^ appearing as bright dots embedded in the grain. (d and e) Close-up HAADF and HRTEM images of the regions marked in (a) and (b), respectively. The clear-cut amorphous rims are marked by the double dashed lines. npFe^0^ is marked by the arrows in (d). (c) EDS map of the region marked in (b). (f) EDS line scan along the dashed line in (c). Both map and line scan show that the outermost rim is rich in O and Si but depleted in Al, Ca, Mg and Fe, and the bright dots are rich in Fe. The EDS line scan shows an unusual enrichment of Al and Ca at the interface between the SiO_x_ amorphous rim and the matrix [marked by the narrow rectangle in (f), corresponding to the bright rim beneath the outermost transparent amorphous rim in (b)]. (g and h) HRTEM images of the marked regions of the inserted glass globules and glass fibers, respectively. Unambiguous deposited amorphous rims can be clearly seen, as marked by the double dashed lines. (i) Deposited rim thickness statistics of the CE-5 samples. Statistics show that rim thicknesses of the CE-5 grains vary from 2.8 to 16.3 nm, with an average of ∼7 nm.

Figure 4 shows the microscopic characters of several representative lunar grains with vapor-deposited rims. As shown in Fig. 4a, b, d and e, both the HAADF and HRTEM images of the glass filament in Fig. 2b clearly present relatively transparent nano-scale amorphous rims coating the surface of the glass matrix. Through characterizations of a series of grains, such surface amorphous rims are found to be common on the CE-5 grains (Fig. 4g and h, Fig. S4 and Fig. S6). As can be seen from Fig. 4d, e, g and h, the unambiguous deposited amorphous rims coat the host grains uniformly with smooth interfaces, indicating that they are formed from vapor atmosphere rather than directional irradiation damage. By comparing the observed numerous amorphous rims, it is found that rim thickness can vary gradually with locations for the same grain (Fig. 4h, Fig. S4e and Fig. S11b) and amorphous rims on different grains can exhibit different characteristic thicknesses (Fig. 4, Fig. S4 and Fig. S6).

Detailed chemical analysis further confirms the vapor deposition origin of such amorphous rims. An EDS map (Fig. 4c) and line scan (Fig. 4f) together determine that the surface rims consist of Si and O as a thin SiO_x_ rim, totally distinguished from the host grain. The deposited amorphous rims on different grains are all found to be rich in Si and O, and depleted in Fe, Mg, Al and Ca (see also Fig. S4 and Fig. S11). Such a compositional disparity independent of host grains demonstrates that the amorphous rims are products of impact-generated thermal vapor deposition processes [8,25]. During thermal vaporization, Si is preferentially vaporized over Fe, Mg, Al and Ca, resulting in enrichment of Si [63]. Nevertheless, the deposited rims on Apollo grains are not pure SiO_x_, but still contain some Fe, Mg, Al and Ca, implying a difference in impact environment between Apollo and the CE-5 landing site. Moreover, the EDS line scan in Fig. 4f finds an unusual enrichment of Al and Ca at the interface between the outermost amorphous SiO_x_ rim and the host glass (see also Fig. S11), exhibiting a bright rim with a thickness of ∼4.8 nm in HAADF images (Fig. 4b and Fig. S11c and f). This element segregation is probably a result of element diffusion from the host grain to SiO_x_ amorphous rims when heated by impacts.

It is noteworthy that the overall thicknesses of impact-generated deposited rims in the CE-5 soils are much smaller than that of Apollo samples. The rim thickness statistics of numerous grains in Fig. 4i show that the thicknesses remain in a narrow range of 2.8–16.3 nm, with the most frequent value of ∼5 nm and an average of ∼7 nm, in stark contrast to Apollo samples, which vary from 10 to 200 nm with an average of 50–60 nm [8,25,27]. It has been demonstrated that the deposited rims are produced by stochastic single impacts rather than accumulation of repetitive micrometeorite impacts [27]. Namely, the deposited rim thicknesses generally depend on the intensity of the corresponding impact events [26,27]. Therefore, the ultra-thin deposited rims here imply an obviously gentler impact environment at the CE-5 landing site compared with Apollo landing sites.

Another significant difference between the deposited amorphous rims of the CE-5 and Apollo samples is the non-existence of npFe^0^ in the CE-5 rims. Extensive investigations of Apollo samples have found abundant npFe^0^ embedded in the deposited amorphous rims [18,20,31], and vapor deposition is proven to be the main process producing npFe^0^ [19,38,64]. The npFe^0^-bearing rims will significantly affect the optical properties of lunar regolith [19,20,31,38]. Unexpectedly, in our studies of numerous CE-5 grains, the deposited amorphous rims contain no npFe^0^ (see typical Fig. 4d, e, g and h, Fig. S4 and Fig. S6), which is very different from previous reports of Apollo samples. Such distinctions are probably because the micrometeorite impacts at the CE-5 landing site are not energetic enough to reach the high temperature required to vaporize and condense Fe elements, consistent with the observed thinner deposited rims herein [26]. Although npFe^0^ is lacking from the vapor-deposited rims of CE-5 grains, npFe^0^ with different sizes is still accumulated inside the grains below the outermost deposited rims, as seen in Fig. 4b and d. This npFe^0^ is probably produced by other space weathering processes such as solar-wind implantations [65], impact-triggered disproportionation reactions [59] and impact-induced thermal decompositions [64], which have been reported in recent researches about CE-5 soils. Moreover, the relatively larger distinct npFe^0^ in more internal positions probably results from the coalescence of tiny npFe^0^ during re-melting, induced by repetitive micrometeorite impacts, like those in agglutinates [19,20,34].

**Figure 5.**
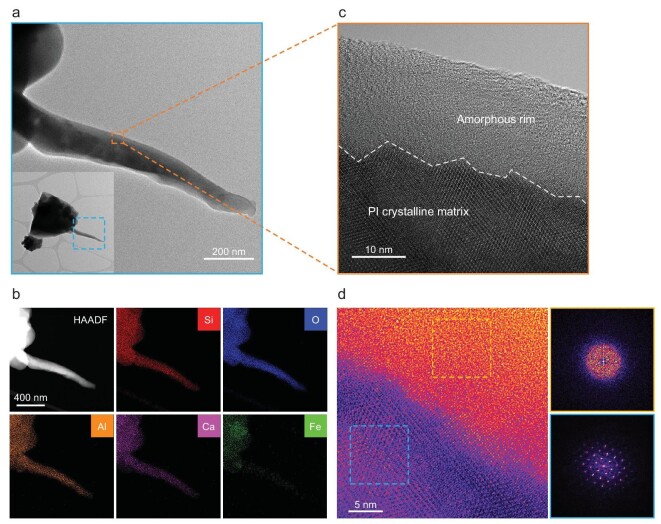
Solar-wind-irradiation-induced amorphous rims. (a) TEM images of a filament of the inserted plagioclase grain. (b) HAADF images and EDS maps of the filament. (c) HRTEM images of the edge of the filament marked in (a). There exists a clear amorphous rim non-uniformly coating the surface of the grain. Distinct from the vapor-deposited amorphous rims, the rim in (c) is not chemically different to the host grain and the interface between the rim and the matrix is sharply serrated (marked by the dashed line), indicating its origin of ion implantation by solar-wind irradiation. (d) HRTEM images of the interface between the amorphous rim and the crystalline matrix. Fast Fourier transforms of the marked rim and matrix regions in (d) confirm their amorphous (top right) and crystalline (bottom right) nature accordingly.

The other kind of surface amorphous rim is induced by the irradiation damage of solar wind. As shown in Fig. 5, there is a clear amorphous rim on the surface of the plagioclase filament. Compared with the vapor-deposited amorphous rims, there is no distinct component difference between the amorphous rim and the host grain, and their interface is sharp and serrated (Fig. 5b–d), demonstrating that the rim is the result of irradiation damage induced by the ion implantations of solar wind [8,29,36]. Energetic ions in solar wind have penetrations of several to tens of nanometers in lunar grains, and implanted ions can gradually damage the crystalline structure along the incidence path, increasing the degree of disorder until there is a fully amorphous damaged layer [28]. It should be mentioned that, in contrast to the deposited amorphous rims produced by random single impact events, the irradiation-induced amorphous rims are positively correlated with the exposure time to solar wind irradiation and have therefore recorded the composition and activities of the Sun [27,31–33].

## DISCUSSION

**Figure 6.**
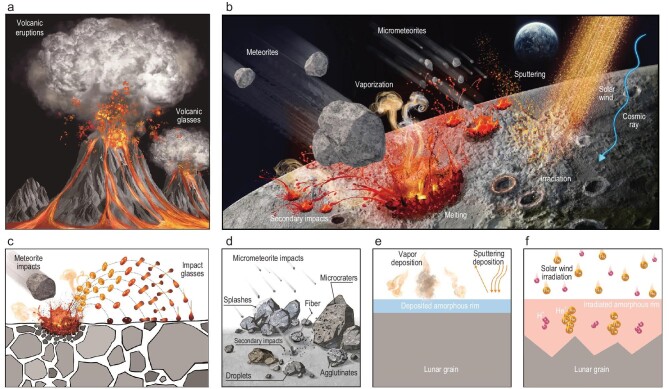
Schematic of the origins of diverse lunar glasses and the corresponding lunar activities. (a) Volcanic eruptions eject fine liquid droplets, forming quenched glass particles called volcanic glasses in pyroclastic deposits. (b) A series of space weathering processes rework the lunar surface, resulting in different kinds of glassy materials. (c) Meteorite impacts melt lunar rocks and eject droplets with different rotations, forming impact glasses with rotational shapes. (d) Frequent micrometeorite impacts cause local melting of lunar rocks, leading to various micron- to nano-scale agglutinate-like, splash-like, droplet-like glasses and ring-like glasses of microcraters, as well as drawing of glass fibers. Debris ejected from a micrometeorite impact crater may hit the surface grains again, resulting in densely distributed craters and additional impact melts. (e) Impact-generated vapor deposition on lunar grains, forming uniform deposited amorphous rims. (f) Solar-wind irradiation damages the surface structures of lunar grains, resulting in non-uniform irradiated amorphous rims. H and He ions in solar wind can be implanted and preserved in the irradiated rims.

Through the systematic collection and characterization of glassy materials in the CE-5 soils, we reveal diverse lunar glasses that have transformed via three main routes, namely, liquid quenching, vapor deposition and irradiation damage of crystalline solids. According to their origins, the lunar glasses can be classified into five types, volcanic glasses, impact glasses, adhered glasses, and deposited and irradiated amorphous rims, which record corresponding activities on the Moon, as illustrated in Fig. 6. Volcanic eruptions on the Moon would produce basaltic lava flows and create fountains of fine molten droplets (Fig. 6a), and then the liquid droplets can be quenched into glass particles, which are dispersed in pyroclastic deposits as volcanic glasses [6]. After the basaltic lavas finally form a mare surface, the lunar surface undergoes continuous space weathering by meteorite bombardment, solar wind and cosmic-ray irradiation (Fig. 6b). Meteorites striking the lunar surface are capable of shattering and melting lunar rocks. The ejected rotational molten droplets will then be quenched during their ballistic flights, forming rotation-featured impact glass particles [47–50,66] (Fig. 6c). Frequent micrometeorite impacts play a pivotal role in reworking lunar regolith by inducing local melting to produce diverse agglutinate-like, splash-like and droplet-like glasses and microcraters altering grain surfaces [1,16,19,61] (Fig. 6d). In addition, when an impactor strikes the lunar surface, the ejected debris may also impact the surface grains again, resulting in densely distributed secondary craters and additional impact melts to produce glass materials [59,60] (Fig. 6d). Furthermore, micrometeorites at hypervelocity are even energetic enough to vaporize lunar materials, resulting in deposited amorphous rims on surface grains [8,19,25–27] (Fig. 6e). Besides impacts, the lunar surface is continuously irradiated by solar wind and cosmic rays without the protection of an atmosphere or magnetic fields. Energetic solar ions will be implanted into grains, damaging the surface crystalline structure to form amorphous rims [8,27–30] (Fig. 6f). These diverse glasses, with the different origins demonstrated here, could be utilized as a catalogue of lunar glasses, providing insights for the further extraction of glass-recorded information.

Among the five kinds of glasses identified in the CE-5 soils, three are related to meteorite impacts, making it possible to constrain the complex impact environment at the CE-5 landing site [2,7]. Most of the glass particles have concentrations of refractory elements similar to that of CE-5 soils, indicating that they are produced from fully molten local regolith generated by large impact events at the landing site [7,51]. Although large meteorite impacts that are capable of producing glass particles are less common at the CE-5 landing site according to previously imaged lower crater density [40], the widespread micron- to nano-scale adhered molten splashes and droplets, vesicular agglutinates, microcraters and deposited amorphous rims explicitly show frequent micrometeorite impacts. The discovery of these multitype glasses, the products of small-scale impacts, shows that the CE-5 soils are heavily reworked by micro-impacts, consistent with the high maturity of the CE-5 soils [40,44].

Besides the heterogeneous flux of meteorites across multiple scales, impact-related glasses can also help elucidate impact velocity [61]. In particular, a distinctive type of impact-generated glass, i.e. glass fibers, are found in the CE-5 soils. The glass fibers can have extreme elongations larger than 50, in contrast to Apollo and Luna grains with elongations generally of 1–3 [1]. Glass fibers are generally drawn from viscous supercooled liquids. To ensure the liquids are able to undergo thermal drawing by external force to form elongated fibers, a high viscosity and corresponding low temperature are required for the molten liquids [57]. The ultra-elongated fibers found in the CE-5 samples therefore suggest obviously gentler impacts that generate melts with a lower temperature and higher viscosity compared to previously explored lunar regions. Furthermore, laboratory studies show that simulant lunar materials can usually be drawn into continuous fibers at 1000–1500°C [54,55]. If lunar glass fibers are assumed to be drawn from melts with a similar temperature, then the corresponding impacts have a rather low velocity of ∼5 km/s, which is much lower than the cosmic velocity of the Moon (15–25 km/s), but close to the average velocity expected for asteroids [1,39,61].

The gentle impact environment is further confirmed by deposited amorphous rims resulting from micrometeorite impacts. The observed widespread deposited rims on the CE-5 grains show features that are distinct from those of Apollo samples [8,18,20,25,27,31]. On the one hand, their thicknesses concentrate on a narrow range of 2.8–16.3 nm with an average of ∼7 nm, much thinner than the average rim thickness of Apollo samples (50–60 nm) [8,25,27]. The ultra-thin thicknesses indicate that the micrometeorite impacts are much gentler and produce less vapor at each single impact event, resulting in much thinner deposited rims. On the other hand, the CE-5 deposited rims are composed of only Si and O, in contrast to Apollo deposited rims, which generally also contain Mg, Al and Ca [8,25,30,67]. This is because Si is more easily vaporized than Mg, Al and Ca, and the less energetic micrometeorites are only enough for SiO_x_ vapors [63]. Particularly, the CE-5 deposited rims contain no npFe^0^, which is generally found in Apollo deposited rims [8,18,20]. According to laboratory simulant experiments [19,26], the vaporization and condensation of npFe^0^ requires impacts with high enough energy density. The common npFe^0^-free deposited rims on the CE-5 grains therefore indicate origins involving gentler impacts with lower velocity than impacts at the Apollo site. These findings collectively indicate that the micrometeorite impacts at the CE-5 landing site are frequent but relatively gentle, in accordance with the low impact velocity reflected by glass fibers.

The above studies of impact-related glasses have preliminarily suggested a micrometeorite-impact-dominant and relatively gentle impact environment at the CE-5 landing site. This distinct impact environment explains the confusion over why the CE-5 soils are highly mature regolith, but have abnormally low glass content [40,44]. The gentle impacts suggest that mechanical brecciation such as fragmentation dominates over melting and vaporization [1,39,61]. The repetitive micrometeorite impacts are thus more efficient at gardening lunar rocks into the very fine grains of mature regolith, than at producing glass materials. Such gentle impacts could further affect space weathering effects. For example, the observed gentle impacts produce ultra-thin vapor-deposited rims without npFe^0^. This finding indicates the minor contribution of impact-generated vapor deposition to the accumulation of npFe^0^ in the observed CE-5 samples and can inspire further investigation into the origins of npFe^0^ beneath the vapor-deposited rims. The expected low meteorite velocity among asteroids has also been found to lead to regolith mixing, resulting in the distinctive space weathering on Vesta [39]. The less abundant melts of gentler impacts may also affect the abundance of lunar surface water, since micrometeorite-impact-generated local melting could promote the reaction of solar-wind implanted H^+^ and lunar materials producing water preserved in agglutinates [12,34]. Therefore, the distinctive impact environment at the CE-5 landing site, and following water content determination, will help us understand the heterogeneous spatial distributions of lunar surface water [24,35].

Besides impact-related glasses, volcanic glasses and irradiated amorphous rims play their own unique roles in recording the Moon's history. In the CE-5 soils, only a small number of the glass particles may be volcanic glasses and they need to be further distinguished in future studies, in combination with other criteria such as major elements homogeneity, absence of exotic inclusions, surface-trapped volatiles and lower ferromagnetic resonance intensity [6]. Although the amount of volcanic glasses is rather low, they have recorded precious information about various volatiles in the lunar mantle [4,10,11,15], which provide crucial insights into the thermal and chemical evolution of the Moon's interior [11], for instance, the longevity of lunar volcanism [41,42], Moon-forming giant impacts [68] and the origins of water and carbon in the Earth–Moon system [4,10,15,41]. The CE-5 volcanic glasses with the youngest ages and highest latitudes among all returned lunar samples thus provide a unique insight into the spatiotemporal evolution of lunar volatiles [9,40]. Finally, the irradiated amorphous rims have recorded information about solar activities and solar-wind materials. Considering the average exposed time and flux of solar wind, almost all surface lunar soils are saturated by solar-wind particles [33]. But limited by the thermal degassing [33], the irradiated amorphous rims on grains in the CE-5 soils from higher latitude regions are prone to preserving more solar components, which are important in the study of the flux variation of solar wind and ^3^He utilization on the Moon [29,32,33]. Meanwhile, the implanted H^+^ from solar wind would take part in a reduction reaction, which could produce npFe^0^ and H_2_O [30,34], resulting in alteration of remotely observed spectra [31,38] and spatiotemporal distributions of surface water [12,24,35,36].

In summary, we conduct a comprehensive morphological, microstructural and geochemical analysis of the CE-5 glassy materials, finding a broad range of lunar glasses with various forms including spherical and rotation-featured particles, ultra-elongated fibers, adhered melts, vesicular agglutinates and surface amorphous rims, produced by volcanic eruptions, meteorite impacts and solar-wind irradiation, respectively. Diverse impact-related glasses have suggested a micrometeorite-impact-dominated and relatively gentle impact environment at the CE-5 landing site. Frequent small-scale impacts play an important role in reworking the CE-5 soils, producing abundant micron- to nano-scale surface alterations. The gentler impact environment results in the diverse distinctions of CE-5 glasses compared with previous lunar samples, including ultra-elongated glass fibers and ultra-thin npFe^0^-free deposited amorphous rims. Besides constraining impact history, each kind of lunar glass has its specific role in recording key information about the Moon. The clarification and classification of diverse lunar glasses provides a guide to further unraveling the many mysteries recorded by appropriate glass types.

## MATERIALS AND METHODS

### Sample preparation

The lunar glasses investigated in this study were from CE-5 lunar soil CE5C0400 allocated by the China National Space Administration. The samples were surficial lunar regolith scooped by the robotic arm of the CE-5 lander in the northeastern Oceanus Procellarum. The samples are fine grains with micron or submicron sizes. They are stored in a glove box protected by dry high-purity nitrogen gas. For each measurement, a minimal dose of soils is extracted from the samples in the glove box.

### Scanning electron microscope analysis

Morphology observations and composition measurements were carried out using a Thermo Scientific Quattro S field emission scanning electron microscope (FE-SEM) equipped with an EDS (Bruker XFlash6|30) detector. Milligrams of samples were directly placed on adhesive carbon-conductive tap carbon foils or carbon-coated copper holders for SEM observations, without any conductive coating. The secondary electron (SE) images were performed at an accelerating voltage of 5–10 kV and an electron beam current of 7–12 pA under high vacuum. The back-scattered electron (BSE) images and EDS analyses were conducted using an accelerating voltage of 15 kV with an electron beam current of 100 pA.

### Transmission electron microscope analysis

The microstructural characterizations were performed on an aberration-corrected JEOL-ARM200F and a JEOL-F200 electron microscope operated at 200 kV. Both of them were equipped with double EDS detectors. Most of the lunar soils were directly placed on carbon-coated copper grids for TEM observations without any extra preparation, avoiding any possible damage or artefacts. The section of the glass dumbbell is prepared by focused ion beam (FIB) cutting using a Talos F200S TEM (Thermo Fisher Scientific). The particle was firstly deposited with Pt for protection. The thin section for TEM experiments was cut from the particle by a 30 kV Ga^+^ ion beam in the FIB system. The section was finally thinned to ∼100 nm using a 10 kV Ga^+^ ion beam and then cleaned using a 5 kV Ga^+^ beam at 40–80 pA. Low ion beam voltage was used in thinning sections to avoid damage or artefacts.

## Supplementary Material

nwad079_Supplemental_File
