# Supplementary material for: Diverse glasses revealed from Chang’E-5 lunar regolith
Source: Natl Sci Rev. 2023 Mar 21;10(12):nwad079. doi: 10.1093/nsr/nwad079 (PMC10632798; doi:10.1093/nsr/nwad079)
Supplement: nwad079_Supplemental_File [file nwad079_supplemental_file.pdf]

Supplementary Data for

Diverse glasses revealed from Chang'E-5 lunar regolith

Rui Zhao[1,2,†], Laiquan Shen[1,†,*], Dongdong Xiao[1,†], Chao Chang[1,2,†], Yao Huang[1,2], Jihao Yu[1,2], Huaping Zhang[1], Ming Liu[4], Shaofan Zhao[4], Wei Yao[4], Zhen Lu[1], Baoan Sun[1,3], Haiyang Bai[1,2,3,*], Zhigang Zou[4,6], Mengfei Yang[4,5], Weihua Wang[1,3,4]

[1]Institute of Physics, Chinese Academy of Sciences, Beijing 100190, China

[2]Center of Materials Science and Optoelectronics Engineering, University of Chinese Academy of Sciences, Beijing 100049, China

[3]Songshan Lake Materials Laboratory, Dongguan, Guangdong 523808, China

[4]Qian Xuesen Laboratory of Space Technology, China Academy of Space Technology (CAST), Beijing 100094, China

[5]China Academy of Space Technology (CAST), Beijing 100094, China

[6]College of Engineering and Applied Sciences, Nanjing University, Nanjing 210093, China

[†]These authors contribute equally to the work.

*Correspondence should be addressed to Laiquan Shen, Haiyang Bai (emails: shenlaiquan@iphy.ac.cn; hybai@iphy.ac.cn).

This PDF file includes:

Supplementary Figures S1 to S11

Supplementary Figures

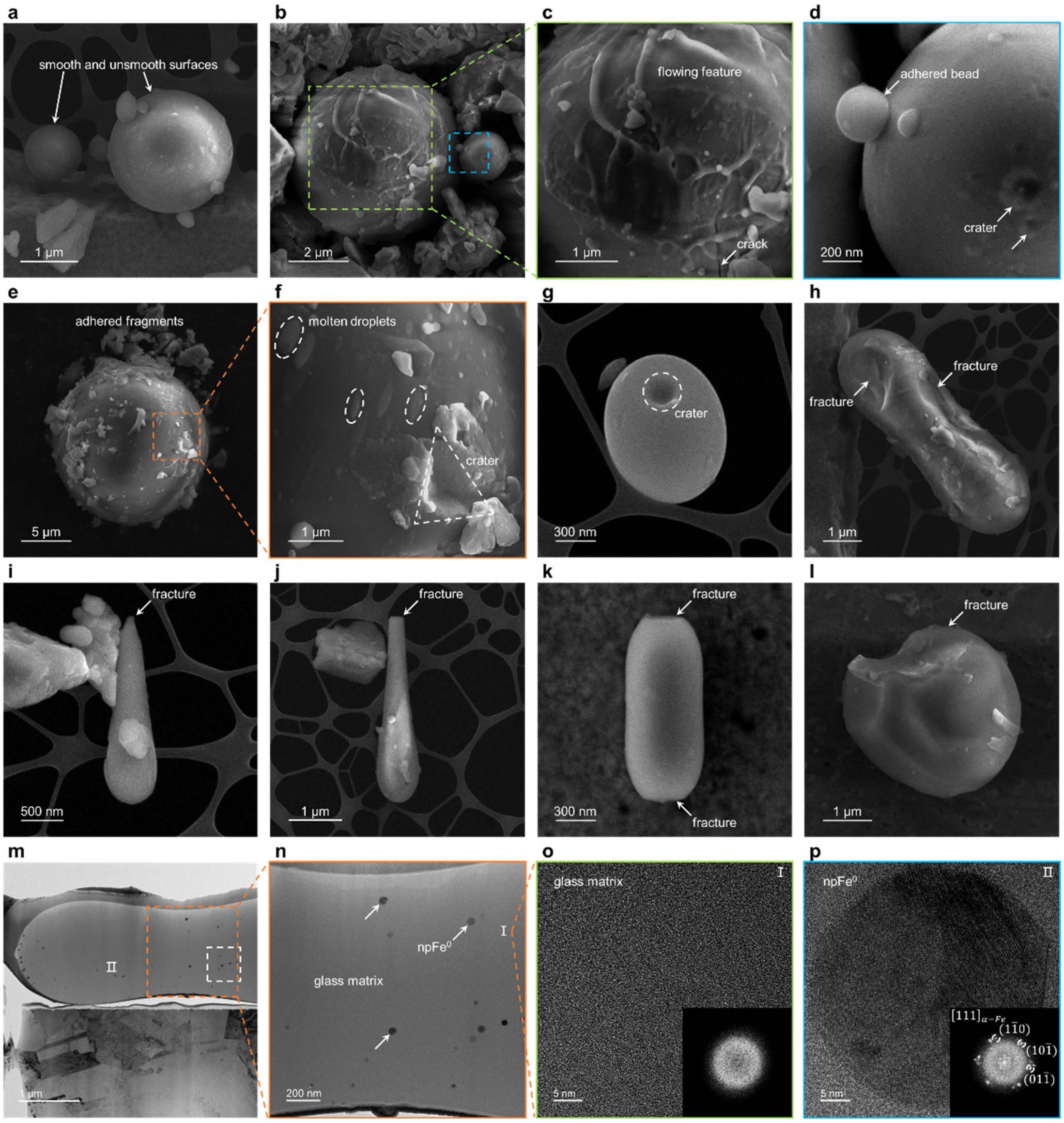

Fig. S1. Scanning electron microscope (SEM) images of morphologies of different glass particles. **a**, Surface morphologies of a smooth (left) and an unsmooth (right) glass globule for comparison. **b**, Surface morphologies of two glass globules with flowing features and adhered objects. **c**, Closeup of the marked region in (**b**). Melting-induced flowing features and a microcrack are found on the glass globule. **d**, Closeup of the marked region in (**b**). Adhered beads and microcraters are observed. **e**, Surface morphology of a glass globule adhered by lots of fragments. **f.** Closeup of the marked region in (**e**)**.** There are adhered molten droplets and a triangular microcrater on the globule surface. **g**, A glass ellipsoid with an isolated microcrater. **h**, A glass dumbbell with brittle fracture surfaces. **i**, **j**, Mace-like glass teardrops with fracture on their tails.

k, A glass dumbbell with fractures on both extremities. **l**, A fractured glass globule. The series of features, including melting-induced flowing, adhered objects, microcraters, microcracks and fractures, all indicate the occurrence of frequent micrometeorite impact events. **m**, Transmission electron microscope (TEM) image of a cross section of a glass dumbbell prepared by focused ion beam (FIB) technology cutting. **n**, Close-up image of the marked region in (**m**). $npFe^0$ (dark dots as indicated by white arrows) are embedded in the glass matrix. **o**, High-resolution TEM (HRTEM) image of the glass matrix. The inset is the corresponding fast Fourier transform (FFT). The HRTEM of the section exhibits typical maze-like structure and the corresponding FFT shows diffuse halos, both confirming the non-crystalline nature of the glass particle. **p**, HRTEM image of the embedded $npFe^0$. The inset is the corresponding FFT along the [111] zone axis of α-Fe.

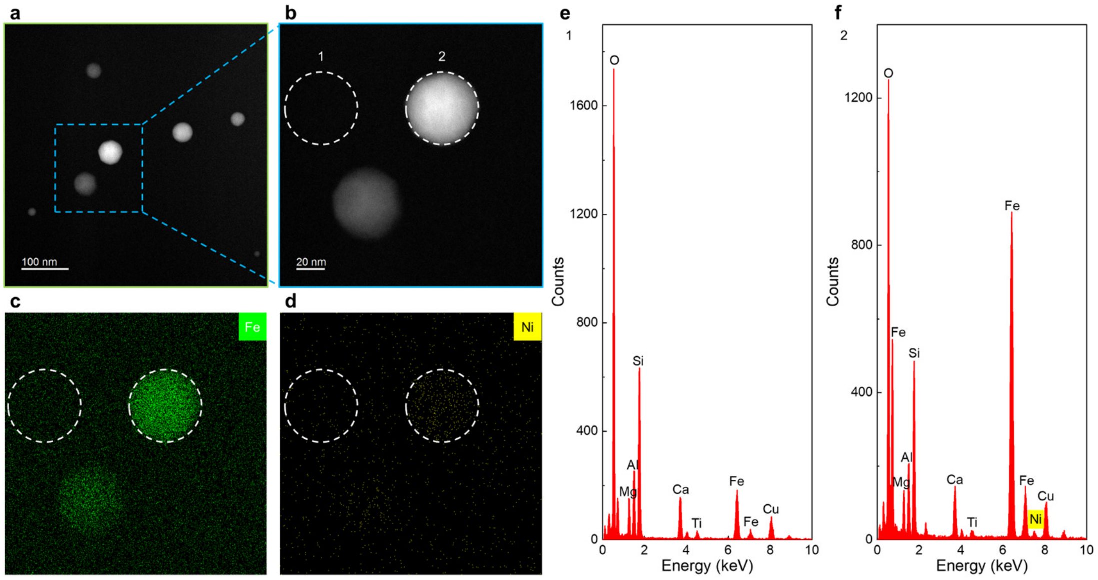

Fig. S2. The Fe-Ni metallic inclusion in the interior of a glass particle. **a**, High-angle annular dark-field scanning transmission electron microscope (HAADF-STEM) image of the region of the FIB section marked by the white box in Fig. S1m. **b**, Close up HAADF image of the marked region in (**a**). The inclusion (marked region 2) exhibits as bright dot in glass matrix. **c**, **d**, Energy dispersive spectroscopy (EDS) maps of the region in (**b**). The elemental distributions indicate that the inclusion is mainly composed of Fe and Ni, and the matrix doesn't contain detectable Ni. **e**, **f**, EDS spectra of the glass matrix (marked region 1) and the inclusion (marked region 2). The peak at 7.47 keV in spectrum of the inclusion is definitely ascribed to be Ni K_α.

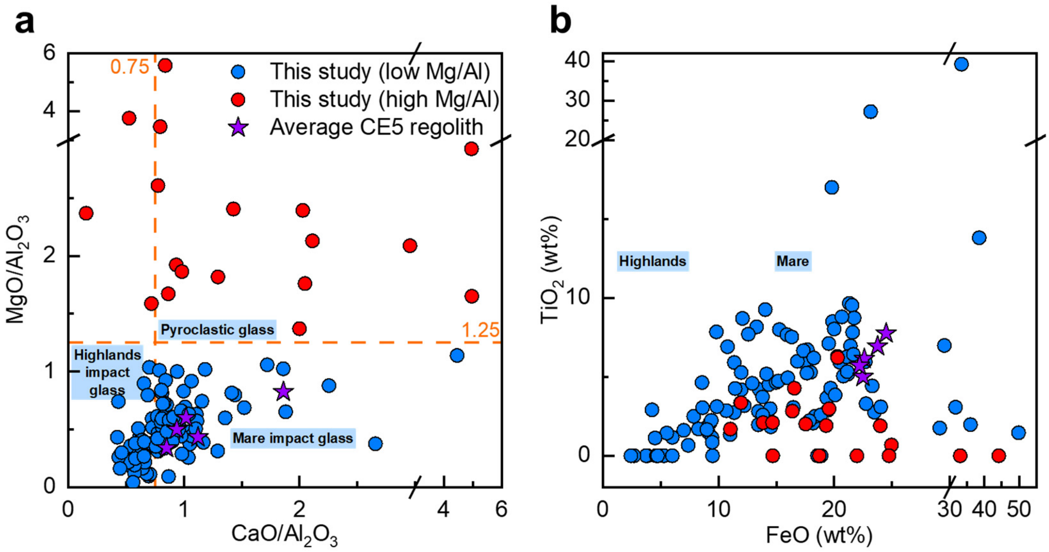

Fig. S3. Two element discrimination diagrams of glass particles. **a**, Major refractory element ratios CaO/Al_2O_3 versus MgO/Al_2O_3. The vertical dashed line highlights the CaO/Al_2O_3 ratio of 0.75 as a dividing line between purely mare and mare-highland mixed glasses. Similarly, the horizontal dashed line highlights a MgO/Al_2O_3 ratio of 1.25 as a dividing line between Apollo pyroclastic and impact glasses [1]. **b**, Elemental variations of TiO_2 versus FeO (wt%). Mare rocks generally have higher FeO [2]. The blue and red dots represent our measurements of glass particles with low Mg content and high Mg content, respectively; the purple pentagrams represent the average compositions of the Chang 'E-5 (CE-5) sampling site reported in the literature [3-6].127 glass particles in the CE-5 soils are shown in the diagram. Most of the glass particles (n=109) can be identified to be impact glasses according to their low MgO/Al_2O_3 ratios. The majority of the impact glasses (n=69) exhibit similar compositions of refractory elements with CE-5 soils, indicating that they are formed by local impact events at the landing site. There are also some impact glasses (n=40) with higher Al content than CE-5 soils. They probably contain exotic materials transported by giant impacts far away from the landing site [7]. Only a minor part of glasses (n=18) shows similar high Mg/Al to Apollo pyroclastic glasses, but whether they are volcanic glasses or exotic impact glasses needs further studies.

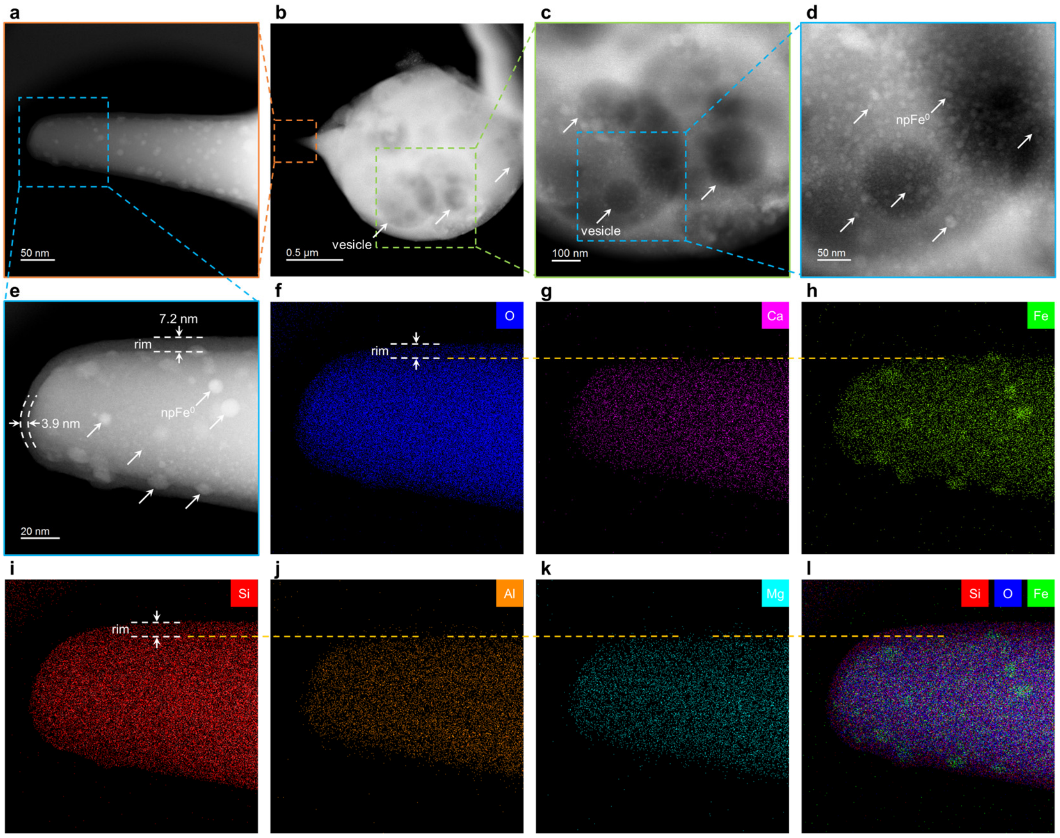

Fig. S4. HAADF-STEM images and EDS maps of the agglutinate glass in Fig. 2a. **a**, **b**, HAADF images of the uniaxial elongated filament and the whole particle, respectively. **c**, **d**, Closeups of the corresponding marked regions in (**b**). It can be seen from the HAADF images that there are large or small rounded vesicles and densely distributed nanophase iron particles (npFe^0) as bright dots in the observed particle, which are typical attributes of agglutinates. **e**, Closeup of the tip of the glass filament. There is an amorphous rim completely coating the filament with the thickness continuously varying from 3.9 to 7.2 nm, as marked by the double dashed lines. Distinct npFe^0 are embedded in the filament beneath the outmost amorphous rim. **f-k**, EDS maps of amorphous rim on the glass filament in (**e**). **l**, An overlay of EDS maps in (**f-k**). The bright dots in HAADF image are further confirmed as npFe^0. The yellow dashed lines in (**f-l**) mark the interface between the amorphous rim and the glass matrix. It can be clearly seen that the amorphous rim is enriched in Si and O, but depleted in Fe, Mg, Al and Ca.

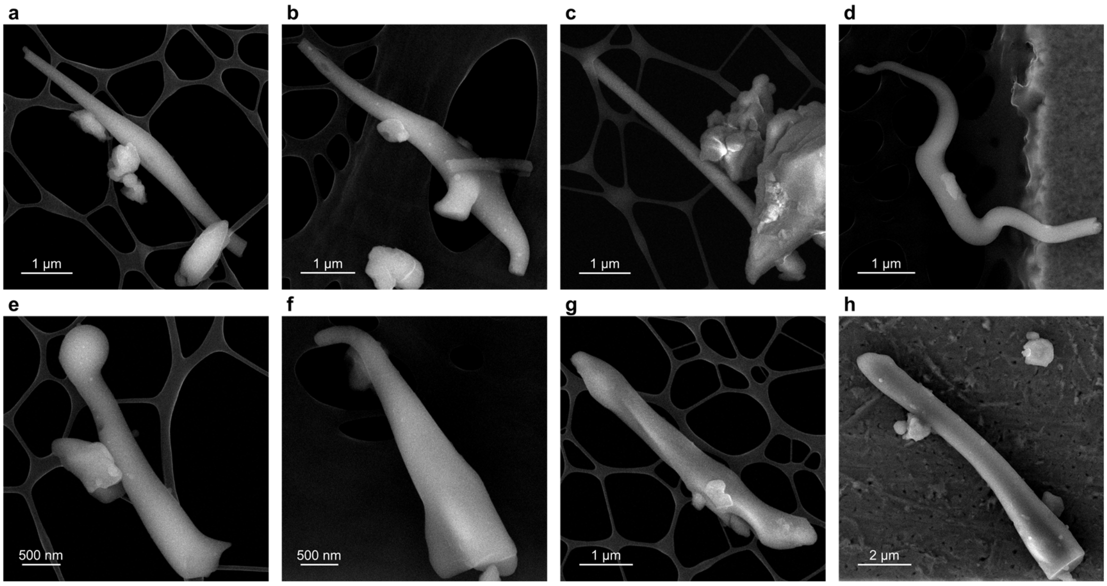

Fig. S5. SEM images of various elongated glass particles. **a**, **b**, Biaxial drawing of two spindle-like glass filaments with decreasing diameter along the drawing directions. **c**, **d**, A straight and a tortuous glass fiber, respectively. **e**-**h**, Four irregular elongated glass rods with different morphologies.

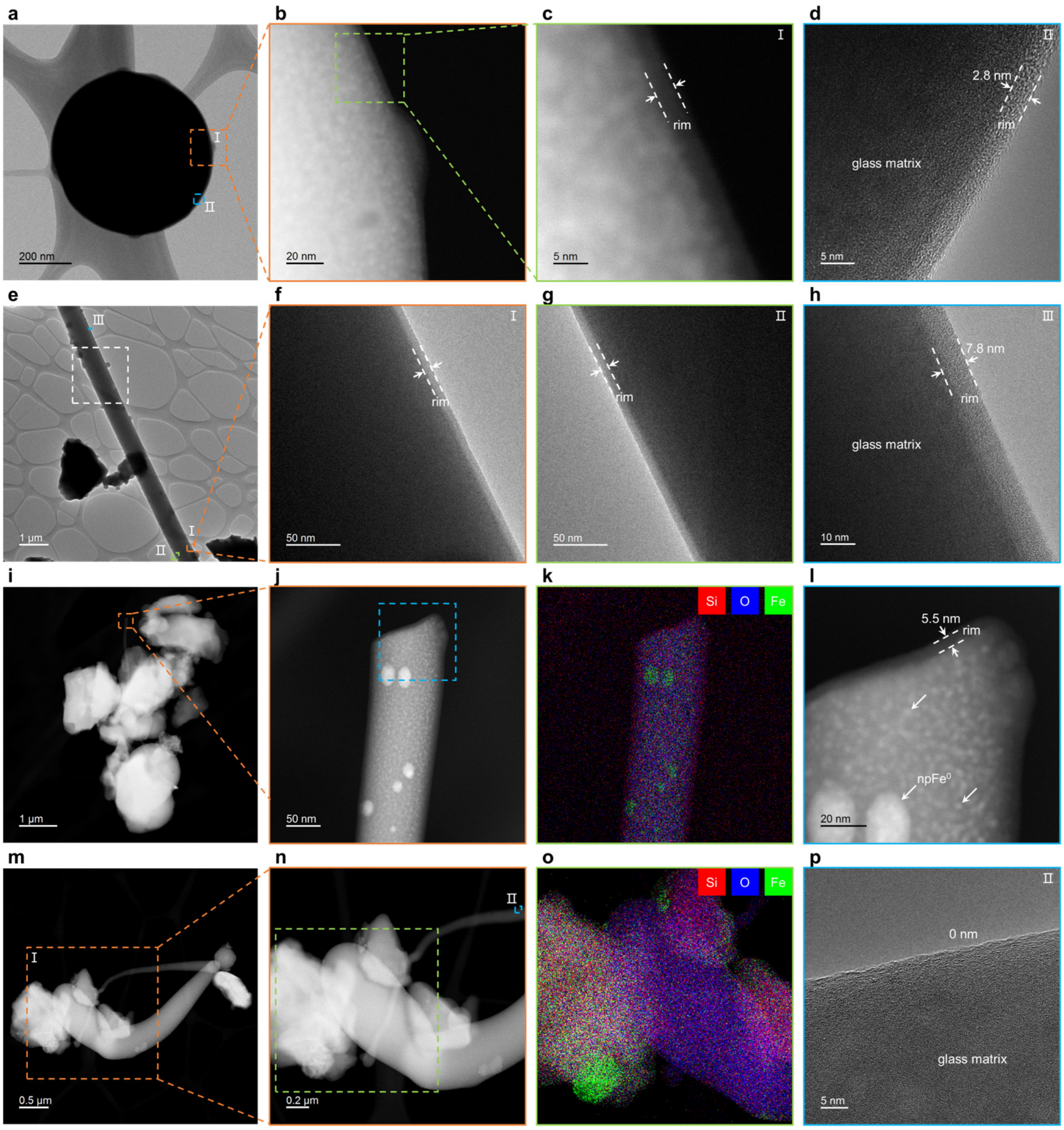

Fig. S6. Microstructures of different glass particles. **a**, TEM image of a glass globule. **b-d**, HAADF and HRTEM images of the marked regions I and II in (**a**). **A** uniform amorphous rim with a thickness of ~2.8 nm can be clearly seen coating the glass matrix of the globule. **e**, TEM image of the glass fiber in Fig. 2g. The white box in (**e**) marks the region of the inserted image in Fig. 5h. **f-h**, Closeups of the marked regions I, II and III in (**e**). Amorphous rim with a thickness of ~7.8 nm can be found at different positions of the fiber, showing a completely coating. **i**, **j**, HAADF images of the fiber in Fig. 2h. **k**, EDS map of (**j**). Dispersed $npFe^0$ are embedded in the glass matrix of the fiber. **l**, Closeup of the marked region in (**j**). Typical characteristics of space weathering, namely, amorphous rims and $npFe^0$ are observed on the surface and in the interior of the natural

lunar glass fiber. **m**, **n**, HAADF images of a glass filament with non-uniform diameter. **o**, EDS map of the marked region in (**n**). **p**, Closeup of the marked region II in (**n**). There are $npFe^0$ in the interior of the glass fiber but no amorphous rim on the surface. The absence of the deposited rims reflects that the grain has not yet experienced micro impact-induced vapor deposition process.

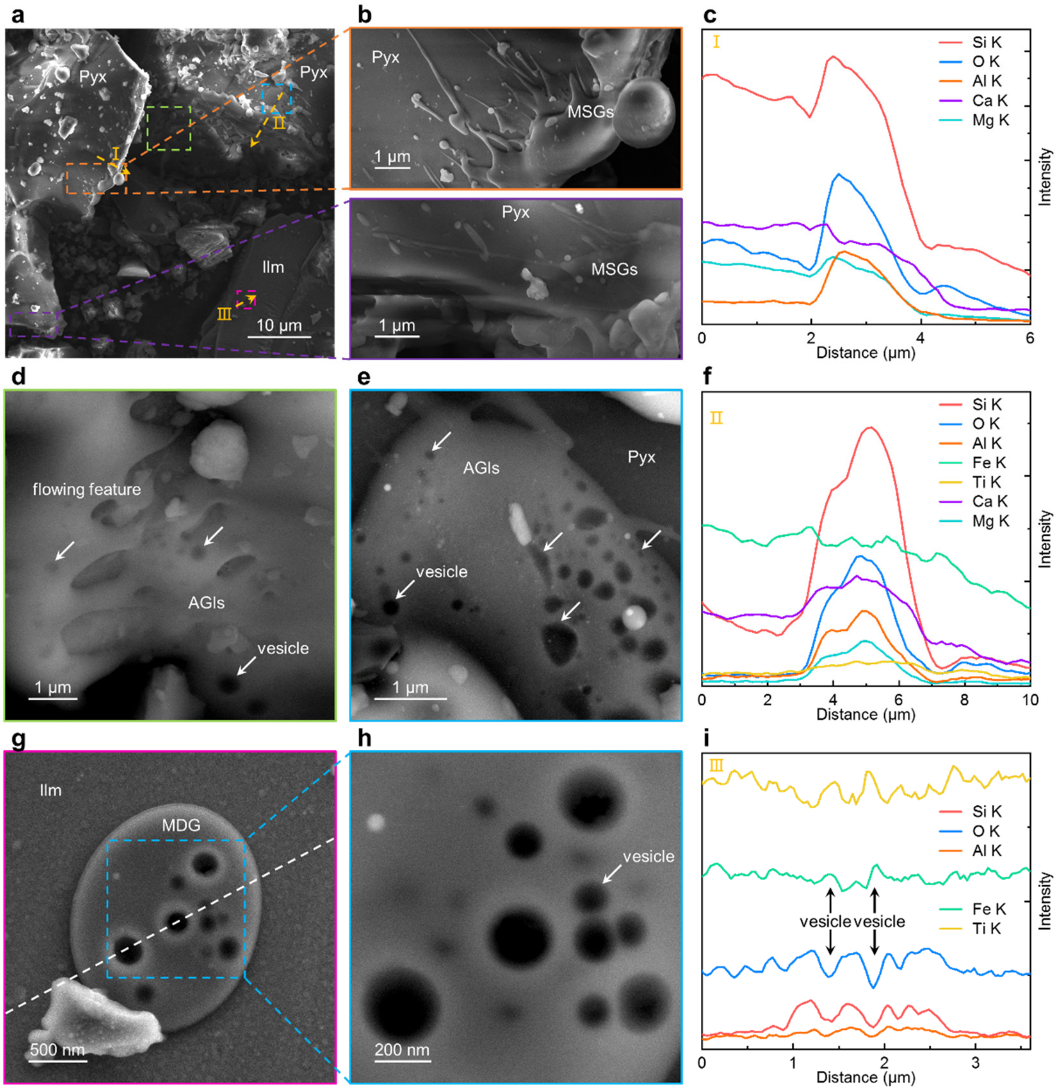

Fig. S7. Supplementary characterizations of the adhered glasses in Fig. 3a. **a**, Secondary electron (SE) image of the grains shown in Fig. 3a. **b**, SE images of the molten splash-like glasses (MSGs) as a melt crashed into the edge of the pyroxene (Pyx) grain. **d**, **e**, Closeup back-scatter electron (BSE) images of the regions marked by the green and blue boxes in (**a**) respectively. The agglutinate-like glasses (AGIs) coating one side of the pyroxene grain exhibit flowing features, and have irregular degassing vesicles and adhered objects. **g**, SE image of the molten droplet-like glass (MDG) in Fig. 3e. **h**, Closeup BSE image of the MDG in (**g**). There are rounded degassing vesicles with sizes ranging from hundreds to tens of nanometers. **c**, EDS line scan along the yellow dashed line I in (**a**). The adhered glasses shown in (**b**) are markedly enriched in O, Si and Al, and are more Mg-rich than the host grain. **f**, EDS line scan along the yellow dashed line

II in (**a**). The AGls are markedly enriched in O, Si, Al and Ca, and are more Mg-rich than the host grain. **i**, EDS line scan along the yellow dashed line III in (**a**), corresponding to the white dashed line across the vesicles of the MDG in (**g**). The MDG is enriched in O and Si, and the vesicle positions show the signal of the host ilmenite (Ilm) grain rich in Fe and Ti. The composition differences between the adhered glasses and their host grains indicate that the AGls, MSGs and MDG are not originated from partially melting of the host grains, but the result of exotic ejected melts induced by micrometeorite impact events.

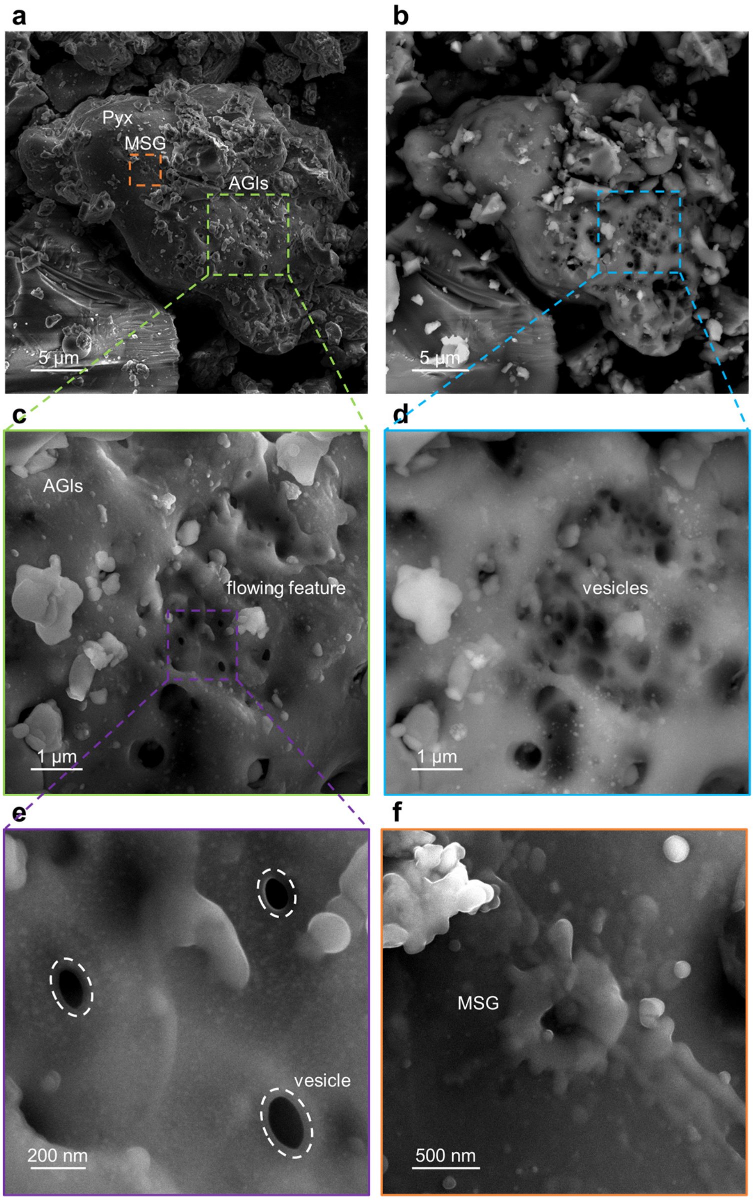

Fig. S8. Morphologies of an agglutinate grain. **a**, **b**, SE and BSE images of an agglutinate grain welded with a pyroxene grain, respectively. The grain exhibits various melting features of a MSG, AGls and adhered objects. **c**, **d**, Closeup SE and BSE images of the AGls marked in (**a**, **b**), respectively. SE image shows the melting-induced flowing features of the surface. Meanwhile, BSE image exhibits the densely distributed vesicles beneath the surface of the AGls. **e**, Typical rounded vesicles of the AGls marked in (**c**). **f**, Closeup SE image of the region marked by the orange box in (**a**). There is an isolated MSG being adhered on the grain surface, showing the crashing of the splashed melt.

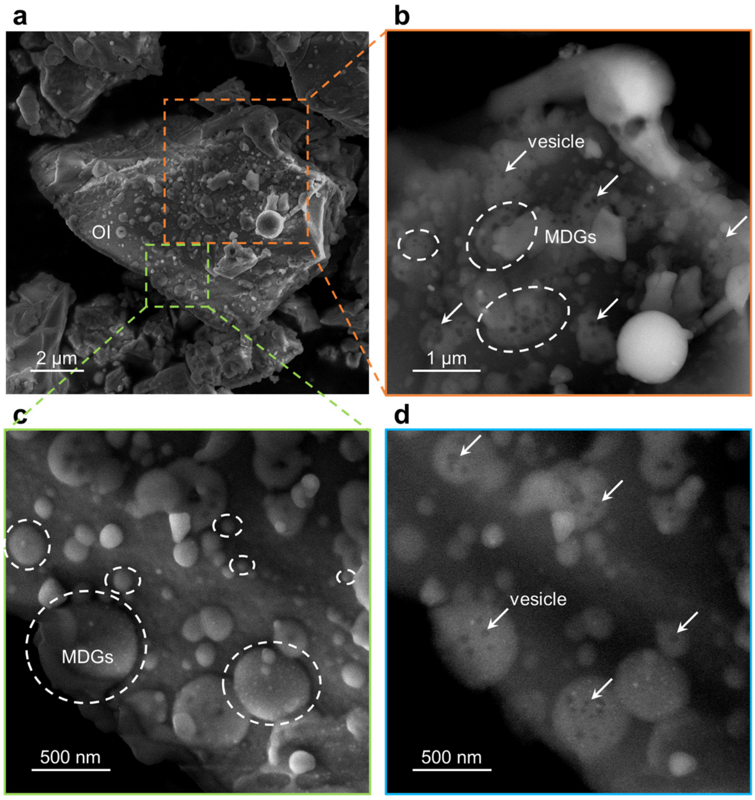

Fig. S9. Supplementary characterizations of the densely distributed adhered MDGs in Fig. 3h. **a**, SE image of the olivine (Ol) grain with adhered glass spheres and densely distributed MDGs. **b**, Closeup BSE image of the marked region in (**a**). The circles mark the adhered glass droplets with different sizes, and the arrows mark the degassing vesicles. **c**, **d**, Closeup SE and BSE images of the marked region in (**a**). SE image shows the densely distributed MDGs with sizes ranging from hundreds to tens of nanometers, as marked by the circles. Meanwhile, BSE image reveals that the MDGs exhibit blistering features with vesicles beneath the surfaces of the glass droplets, as marked by the arrows.

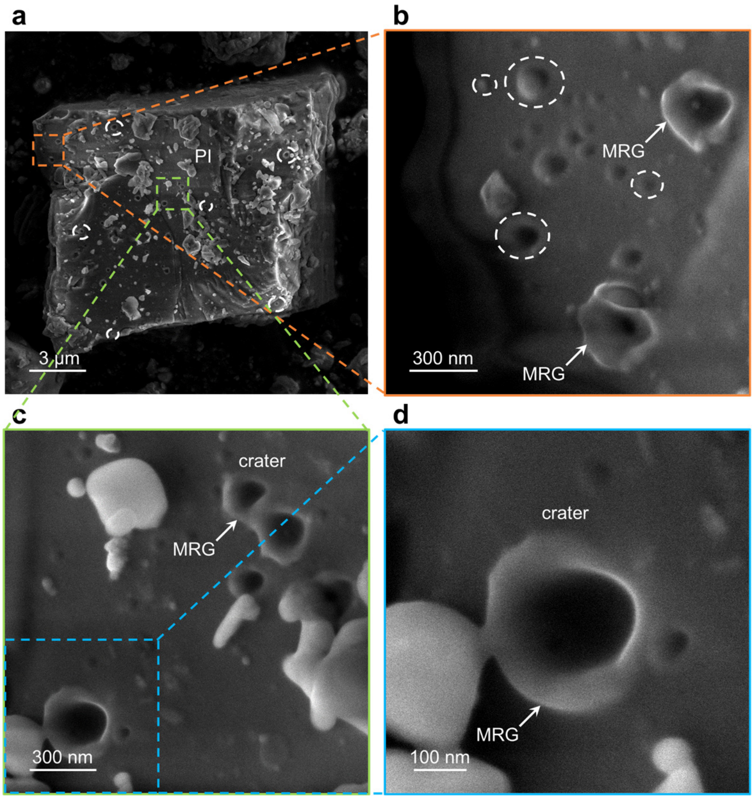

Fig. S10. Supplementary characterizations of microcraters on the surface of the grain in Fig. 4. **a**, SE image of the plagioclase (Pl) grain. The grain has sharp fracture surfaces, and the upper surface is full of craters, as marked by the circles. **b-d**, Closeups of different regions of the grain surface marked in (**a**). There are craters with different sizes and raised molten ring-like glasses (MRGs) overhanging the edge of the craters spreading all over the grain surface. The variation of morphologies of the glass rings might probably results from micro impacts with different incidence angles.

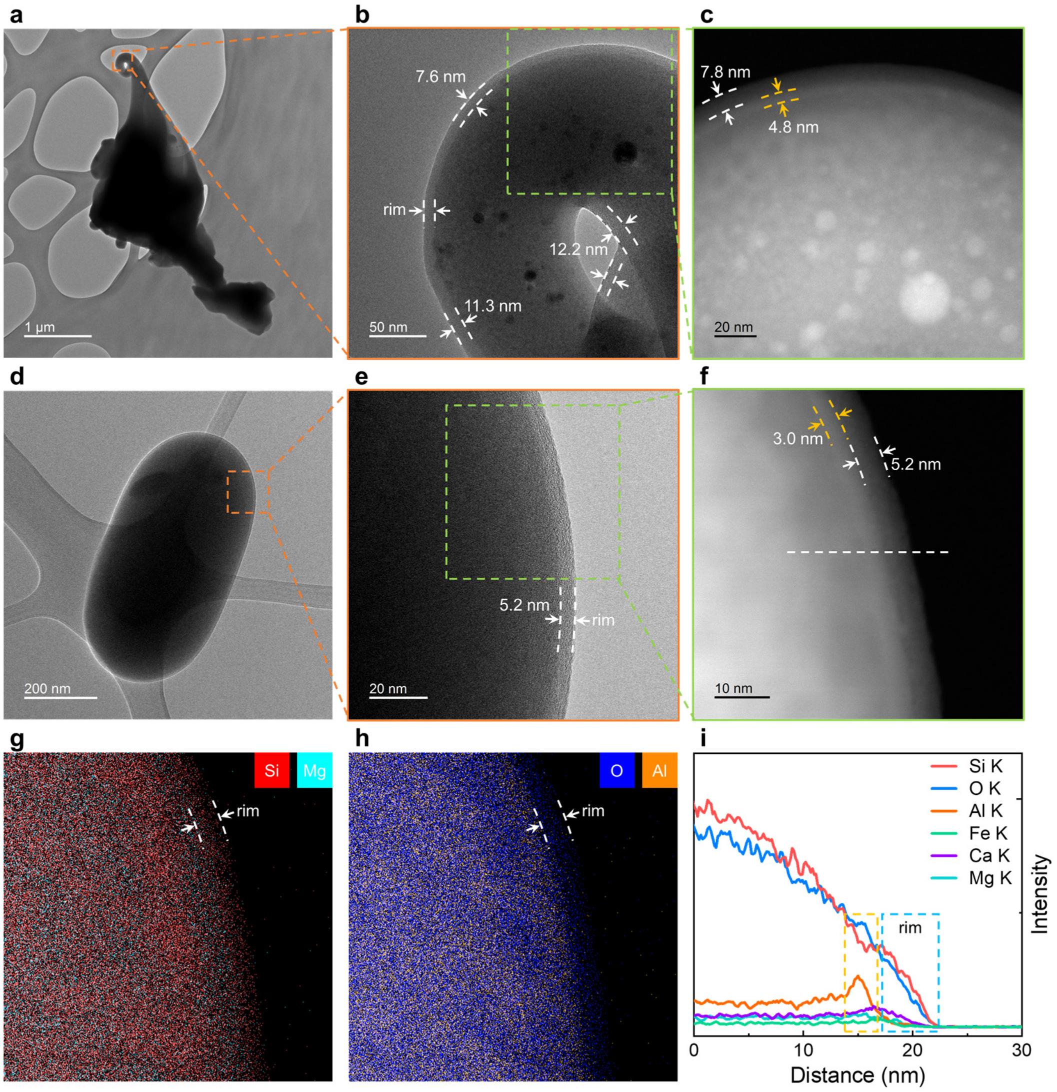

Fig. S11. Supplementary characterizations of vapor deposited amorphous rims in Fig. 5. **a**, TEM image of the whole particle with twisted filament in Fig. 2b and Fig. 5a. **b**, Closeup of the marked region in (**a**). Thickness of the amorphous rim coating the grain surface varies gradually along the curved surface, as marked by the white double dashed lines. Dispersed $npFe^0$ appearing as dark dots are embedded in the glass matrix beneath the amorphous rim. **c**, HAADF image of the marked region in (**b**). There is a bright rim between the outmost amorphous rim and the host matrix, as marked by the yellow double dashed lines. **d**, TEM image of the glass globule inserted in Fig. 5g. **e**, Uniform amorphous rim with a thickness of ~5.2 nm coating the globule completely. **f**, HAADF image of the marked region in (**e**). The bright rim between the outmost amorphous rim and the matrix can be also observed, as marked by the yellow double dashed lines. **g**,

h, EDS maps of the region in (**f**). **i**, EDS line scan along the white dashed line in (**f**). Maps and line scan both show that the outmost rim is enriched in Si and O, but depleted in Fe, Mg, Al and Ca, consistent with the results of different grains in Fig. S4 and Fig. 5. The bright interface rim (marked by the yellow box) is enriched in Al and Ca, which is probably induced by elements diffusion between outmost rim and the matrix.

References

1. Zeigler RA, Korotev RL, Jolliff BL *et al.* The geochemistry and provenance of Apollo 16 mafic glasses. *Geochim Cosmochim Acta* 2006; **70**(24): 6050-67.
2. Reid A, Ridley W, Warner J *et al.* Chemistry of highland and mare basalts as inferred from glasses in the lunar soils. In: *3rd Lunar and Planetary Science Conference, 1972*.
3. Li CL, Hu H, Yang MF *et al.* Characteristics of the lunar samples returned by the Chang'E-5 mission. *Natl Sci Rev* 2022; **9**(2): nwab188.
4. Zhang H, Zhang X, Zhang G *et al.* Size, morphology, and composition of lunar samples returned by Chang'E-5 mission. *Sci China Phys, Mech Astron* 2022; **65**(2): 1-8.
5. Che XC, Nemchin A, Liu DY *et al.* Age and composition of young basalts on the Moon, measured from samples returned by Chang'e-5. *Science* 2021; **374**(6569): 887-90.
6. Tian HC, Wang H, Chen Y *et al.* Non-KREEP origin for Chang'e-5 basalts in the Procellarum KREEP Terrane. *Nature* 2021; **600**(7887): 59-63.
7. Zellner NEB and Delano JW. $^{40}Ar/^{39}Ar$ ages of lunar impact glasses: Relationships among Ar diffusivity, chemical composition, shape, and size. *Geochim Cosmochim Acta* 2015; **161**: 203-18.
